# Effect of a Single and Triple Dose of Levamisole on Hematological Parameters in Controlled Inflammation Model

**DOI:** 10.3390/ani12162110

**Published:** 2022-08-17

**Authors:** Piotr Kuropka, Anna Leśków, Katarzyna Małolepsza-Jarmołowska, Maciej Dobrzyński, Małgorzata Tarnowska, Jacek Majda, Maciej Janeczek, Katarzyna Żybura-Wszoła, Andrzej Gamian

**Affiliations:** 1 Department of Biostructure and Animal Physiology, Wrocław University of Environmental and Life Sciences, Norwida 31, 50-375 Wrocław, Poland; 2 Department of Basic Sciences, Wroclaw Medical University, Chałubińskiego 4, 50-368 Wrocław, Poland; 3 Department of Drug Form Technology, Wroclaw Medical University, ul. Borowska 211A, 50-556 Wrocław, Poland; 4 Department of Pediatric Dentistry and Preclinical Dentistry, Wroclaw Medical University, Krakowska 26, 50-425 Wrocław, Poland; 5 Department of Laboratory Diagnostics, 4th Military Hospital, Weigla 5, 50-981 Wrocław, Poland; 6 Hirszfeld Institute of Immunology and Experimental Therapy, Polish Academy of Sciences, Weigla 12, 53-114 Wrocław, Poland

**Keywords:** morphology, blood parameters, inflammation, pleuritis

## Abstract

**Simple Summary:**

The inflammatory reaction in the body is a very complex process linked with a series of changes in many parameters, including hematological ones. Levamisole is a broad-spectrum anthelmintic used in veterinary medicine. This antiparasitic agent is furthermore suspected of immunomodulatory activity. Therefore, this study is focused on the immunostimulating effect of single and triple doses of levamisole on hematological parameters. During the experiment, it was observed how levamisole modified the inflammatory response to carrageenan in the rat pleural cavity. This study showed the immunomodulatory effect of levamisole on the blood parameters and mild reduction in pneumonia in histopathological examination.

**Abstract:**

This study aimed to evaluate the impact of single and triple administration of levamisole on the dynamics of hematological parameters during experimental pleuritis. The experiment was performed on female Buffalo rats. Rats were randomly assigned to two equal groups that received 1 and 3 doses of levamisole every 2, 24 and 48 h, respectively. Following the experiment, blood samples for the measurement of hematological parameters were collected. The study group receiving three doses of levamisole observed a significant reduction of red blood cell count at 48 h post administration and an increase in mean corpuscular volume compared to the control inflammation group. The administration of a single dose of levamisole results in a significant increase in hematocrit at 72 h, an increase in white blood cell count at 24 h and 72 h, and an increase in neutrophil count at 72 h compared to the control inflammation group. Administration of a single and triple dose of levamisole showed statistically significant modification of some hematological parameters and thus modulates the inflammatory process. In the lungs, this results in a reduction in leukocyte infiltrations around the bronchi and blood vessels.

## 1. Introduction

Levamisole (LMS), a left-handed form of tetramisole, is an antibiotic belonging to the group of imidothiazoles. It was discovered in 1960 as a veterinary drug with anthelmintic activity [1,2,3,4]. Levamisole is a selective agonist of nicotinic acetylcholine receptors [5,6,7,8].

At present, the ongoing studies are mainly focused on the immunostimulating effect of levamisole. It has been used in the treatment of chronic infections, cancers [5], and autoimmune diseases, including rheumatoid arthritis, ankylosing spondylitis, systemic lupus erythematosus, nephrotic syndrome, and vitiligo, as well as in the therapy of warts and hepatitis B virus infection [1,3,9,10,11,12]. The effect of levamisole on the immune system is not fully known; however, this drug is successfully used to prolong remission in children with steroid-dependent nephrotic syndrome, in the treatment and prophylaxis of atopic skin lesions, and in other severe diseases manifesting with cutaneous lesions, inter alia leprosy [13].

Levamisole contributes to the initiation of the local humoral response against intestinal pathogens, such as enterotoxigenic *Escherichia coli*. This drug is used as an adjuvant to a vaccine against post-weaning *E. coli* infection in pigs; this vaccine induces clinical improvement in the vaccinated animals and is important in immunoprophylaxis of enteric colibacillosis [14].

Previous studies on levamisole are focused on its effect on immune response and on using this response in anti-cancer regimens. Levamisole combined with 5-fluorouracil (5-FU) is an effective adjuvant for chemotherapy of some types of cancers, such as stage III colon cancer; however, it is ineffective [7,12,13] in rectal cancer [9,15,16,17].

Levamisole exhibits cytotoxic activity. Studies on the mechanism of the above-mentioned effect demonstrate that this drug induces apoptosis of myeloma cells through an increase in DNA fragmentation, release of cytochrome c to the cytoplasm, and stimulation of caspase-3 activity in cells. This suggests that levamisole can be a strong agent in the therapy of multiple myeloma [18,19].

In addition, levamisole is a strong inhibitor of mammalian alkaline phosphatase (ALP) activity (EC 3.1.3.1) [18,20,21]. High levels of ALP activity were observed in different types of malignant cells as well as in serum samples taken from cancer patients [22]. Moreover, high levels of enzyme expression were also observed in cancer cell lines. Levamisole administered at concentrations of 0.1 and 0.5 mM inhibited more than 80% of ALP activity (non-competitive inhibition) [20,21]. It also inhibits the ALP activity and proliferation of lipopolysaccharide-stimulated murine B-cells [18]. ALP inhibition seems to be important in the reduction of pathology during the course of *Plasmodium falciparum* infection due to a decrease in the number of adherent infected red blood cells (iRBs).

Purzyc and Calkosinski assessed the in vivo effect of levamisole on ecto-ATPase activity in B- and T-cell populations in induced pleuritis. The above-mentioned study has demonstrated that levamisole reduces ecto-ATPase activity in both lymphocyte populations [23].

In rats immunized with a sheep red blood cell (SRBC) suspension, the in vivo and in vitro effect of levamisole on ecto-ATPase activity in B- and T-cells populations was also evaluated. Levamisole was administered at a dose of 2.5 mg/kg bw in two regimens—one and four doses. In vitro studies of levamisole have not demonstrated any effect on ecto-ATPase activity; however, during in vivo administration, inhibitory effects on ecto-ATPase activity, both after single and quadruple administration, were observed [24].

Levamisole administered to rats immunized with SRBC suspension at a dose of 2.5 mg/kg bw increased the primary humoral response against sheep RBC. In addition, it increased the number of plaque-forming cells (PFCs) and the level of anti-SRBC antibodies [25]. Furthermore, the effect of levamisole on dioxin receptors expression and glycation in a chicken embryo model was examined [26].

Until now, there have been no studies to assess the effect of levamisole on inflammation in the context of immunomodulatory effects. The aim of the paper was to assess the impact of single (at 2 h) and triple (at 2, 24, and 48 h) administration of levamisole at a dose of 2.5 mg/kg bw in rats with experimentally induced pleuritis. The current study performed in a rat model of inflammation demonstrates the immunomodulatory properties of levamisole as well as its effect on the dynamics of hematological parameters.

## 2. Materials and Methods

### 2.1. Experimental Animals

Experiments were performed on Buffalo rats taken from the animal farm of the Wroclaw Medical University. These experiments were performed on females aged 8–10 weeks, weighing 120–140 g; rats were kept in air-conditioned rooms (temperature: 21–22 °C, humidity: 62–63%) on a standard Murigran diet with unlimited access to water. Experimental rats were kept in standard polystyrene cages, six animals each. The study was approved by the Local Ethics Council for Animal Experiments (Permission number: 83/2012).

### 2.2. Experimental Pleuritis Model

Experimental pleuritis was induced in rats by intrapleural injection of 0.15 mL of 1% carrageenan solution buffered in saline (Sigma-Aldrich, Burlington, MA, USA) into the right 4–5th intercostal space at the level of the elbow joint. In the control group, carrageenan solution was replaced with buffered saline.

Rats injected with an aqueous 10% levamisole (Vetoquinol Biowet, Gorzów Wielkopolski, Poland) at a dose of 2.5 mg/kg bw were divided into two study groups (LMS I dose and LMS III dose). The first group (LMS I dose) received an intraperitoneal injection (IP) of levamisole at a single dose of 2.5 mg/kg bw 2 h after carrageenan administration, while the second group (LMS III dose) received three intraperitoneal doses of levamisole at 2, 24 and 48 h post carrageenan injection. In all study groups, blood samples were drawn at 24, 48 and 72 h post carrageenan injection. The experimental groups of in vivo experiment were as follows:

1. IP—Control inflammation—18 females. Rats received 0.15 mL of 1% of carrageenan solution intrapleurally. Blood samples were collected at 24, 48, and 72 h from baseline.
2. LMS I 24—6 females. Induction of pleuritis with subsequent single administration (IP) of levamisole at a dose of 2.5 mg/kg bw, 2 h after inflammation induction. Blood samples were collected at 24 h from baseline.
3. LMS I 48—6 females. Induction of pleuritis with subsequent single administration (IP) of levamisole at a dose of 2.5 mg/kg bw 2 h after inflammation induction. Blood samples were collected at 48 h from baseline.
4. LMS I 72—6 females. Induction of pleuritis with subsequent single administration (IP) of levamisole at a dose of 2.5 mg/kg bw 2 h after inflammation induction. Blood samples were collected at 72 h from baseline.
5. LMS II 48—6 females. Induction of pleuritis with subsequent double administration (IP) of levamisole at a dose of 2.5 mg/kg bw 2 and 24 h after inflammation induction. Blood samples were collected at 48 h from baseline.
6. LMS III 72—6 females. Induction of pleuritis with subsequent triple administration (IP) of levamisole at a dose of 2.5 mg/kg bw 2, 24 and 48 h after inflammation induction. Blood samples were collected at 72 h from baseline.

### 2.3. Blood Sample Collection and Hematological Analysis

Rats underwent barbiturate anesthesia before collecting biological material for testing. Pentobarbital (Biochemie GmbH, Hamburg, Germany) was injected intraperitoneally at a dose of 30 mg/kg bw. Next, the abdominal cavity was opened, and blood samples were collected with the use of a 0.8 mm needle into standardized hematological tubes with potassium-EDTA and into blood serum tubes (Sarstedt, Nümbrecht, Germany). Animals were sacrificed immediately after blood samples were collected. Blood samples were evaluated with the use of a Sysmex XT-1800i hematology analyzer (Sysmex Poland Ltd., Warszawa, Poland).

In blood serum, the following parameters were measured: white blood cells (WBC), red blood cells (RBC), hemoglobin (HGB), hematocrit (HCT), mean corpuscular volume (MCV), mean corpuscular hemoglobin (MCH), mean corpuscular hemoglobin concentration (MCHC), red blood cell distribution width (RDW), hemoglobin concentration distribution width (HDW), mean platelet volume (MPV), platelet distribution width (PDW), plateletcrit (PCT), neutrophils (NEUT), lymphocytes (LYM), monocytes (MONO), eosinophils (EOS), basophils (BASO), large unstained cells (LUC), and platelet count (PLT) [27].

### 2.4. Histological Analysis

Immediately after rats were sacrificed, histological material was collected and prepared for histological analysis. Lung samples were fixed in a 4% water solution of buffered formalin (pH 7.2–7.4) for 24 h. The material was rinsed in running water, dehydrated in alcohol series and embedded in paraffin. Sections 7 µm thick were stained with hematoxylin and eosin. The material was analyzed and documented with a Nikon Eclipse 80i (Nikon, Tokyo, Japan) microscope by NIS-Elements Ar 4.00 software (Nikon, Tokyo, Japan).

The material for histological examination was taken from animals after: IP—72 h, LMS I-24—24 h, LMS I-48—48 h, LMS I-72—72 h, LMS II-48—48 h, and LMS III-72—72 h.

### 2.5. Statistical Analysis

Values of hematological parameters in the blood of rats were analyzed with the use of Statistica 13.0 software (StatSoft Ltd., Kraków, Poland). The arithmetic mean of the analyzed parameters (x) for the specified number of studied animals (N) was determined, as well as the standard deviation (SD) and minimal (MIN) and maximal (MAX) values of parameters. Once the normal distribution of data was confirmed, groups LMS I and LMS III were compared to IP group with the use of Student’s t-test with Bonferroni correction in order to determine the level of statistical significance. Differences between groups were considered significant when *p* ≤ 0.05.

With regard to the selected parameters (WBC, RBC, HCT, HGB, NEUT, LYM, PLT), a correlation analysis (Pearson’s r) at a 95% confidence interval (CI) was performed. Assuming that correlation coefficients are significant when the *p*-value is <0.05, an analysis of results’ significance levels for the above-mentioned parameters was also conducted.

## 3. Results

### 3.1. Red Blood Cell System

After 48 h, we observed a statistically significant reduction of RBC count in the LMS II 48 group compared to the IP group (*p* = 0.04). A single dose of levamisole increases hemoglobin (*p* = 0.01) and hematocrit level (*p* = 0.04) at 72 h after administration compared to values obtained in the IP group within the same period of time. Triple-dose administration of levamisole results in a decrease in RBC count, hemoglobin, and hematocrit levels at 48 h and 72 h. After 48 h of inflammation and two doses of levamisole administered (LMS II 48 h), RBC and HGB are significantly decreased in comparison to levels measured for the IP group (*p* = 0.04, *p* = 0.02, respectively). Data obtained for LMS III 72 h, in this case, are not statistically significant; therefore, this trend we observe should be under further examination.

**Figure 1:**
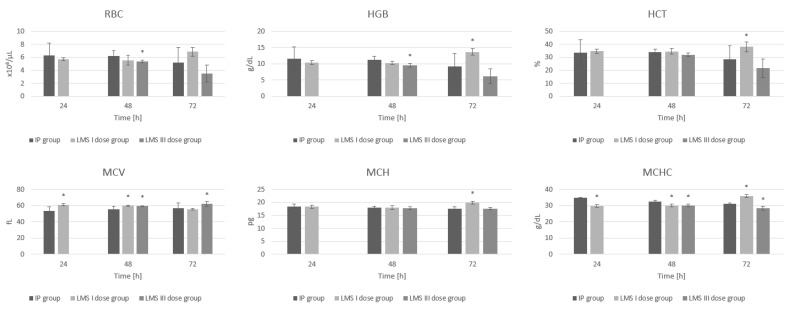
The influence of LMS in single and triple application on the red blood cells (RBC), hemoglobin (HGB), hematocrit (HCT), mean corpuscular volume (MCV), mean cell hemoglobin (MCH), mean corpuscular hemoglobin concentration (MCHC)—parameters measured during experimentally induced pleuritis in rats. *: *p* < 0.05 vs. IP group.

The administration of a single dose of levamisole contributes to an increase in MCV at 24 h and 48 h (*p* < 0.001 and *p* = 0.01 respectively), MCH at 72 h (*p* < 0.001), and MCHC at 48 h and 72 h (both *p* < 0.001) compared to IP group. In a group receiving three doses of levamisole, statistically significant differences in MCV values at 48 h (LMS II 48, *p* = 0.048) and 72 h (LMS III 72 h, *p* = 0.05) compared to the control inflammation group were demonstrated (Figure 1). All results are included in Table S1 (as Supplementary Data).

### 3.2. White Blood Cell System

**Figure 2:**
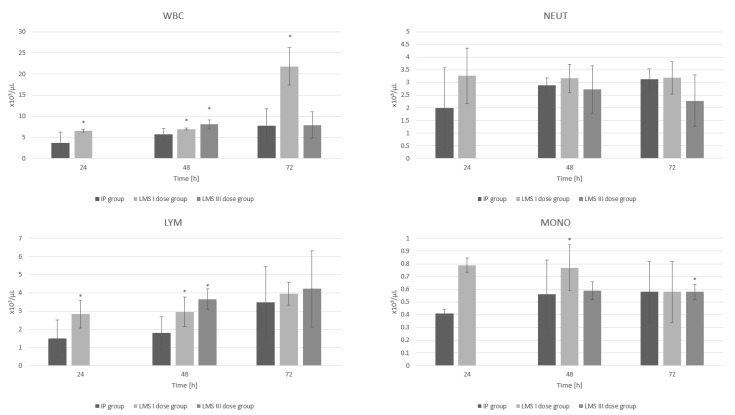
The influence of LMS of single and triple applications on white blood cells (WBC), neutrophils (NEUT), lymphocytes (LYM and monocytes (MONO) during experimentally induced pleuritis in rats. *: *p* < 0.05 vs. IP group.

In the LMS I group, the overall white blood count (WBC) contributes to a statistically significant increase in the number of these morphotic elements at 24 h, 48 h and 72 h compared to the IP group (*p* = 0.03, *p* = 0.05 and *p* < 0.001, respectively). In the LMS III group, a significant increase in WBC at 48 h (*p* = 0.01) compared to the IP group is also observed (Figure 2).

A single-dose administration of levamisole increases lymphocyte count at 24 h and 48 h (*p* = 0.02 and *p* < 0.001, respectively) in the experimental group compared to the IP group. Moreover, administration of two doses of levamisole increases lymphocyte count at 48 h (LMS II 48 *p* < 0.001) compared to the IP group (Figure 2).

Considering the very large standard deviations of the neutrophil count measurement results, it is not possible to determine with certainty a single trend of levamisole’s influence on this parameter. However, single-dose administration of levamisole tends to increase the neutrophil count at 72 h compared to the control inflammation group, while administration of three doses of levamisole results in a decrease in the neutrophil count at the same time point compared to the control inflammation group (Figure 2). All results are included in Table S1 (as Supplementary Data).

### 3.3. Platelets

After single-dose administration of levamisole, a statistically significant increase in platelet count at 72 h (*p* = 0.01) of inflammation compared to the IP group is observed. Moreover, the LMS III group demonstrates a significant decrease in platelet count (*p* = 0.02, *p* < 0.001) and plateletcrit (*p* = 0.02, *p* < 0.001) at 48 h and 72 h of the experiment compared to the same time points in the group with induced inflammation.

**Figure 3:**
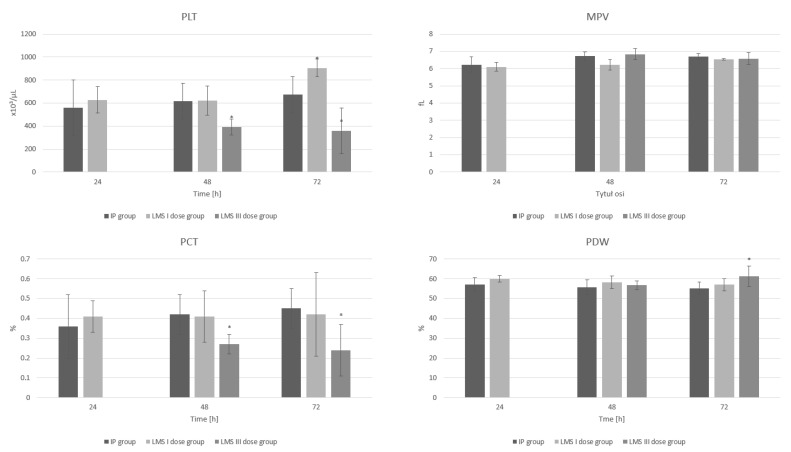
The influence of LMS in single and triple application on the platelet (PLT), mean platelet volume (MPV), plateletcrit (PCT), and platelet distribution width (PDW) parameters during experimentally induced pleuritis in rats. *: *p* < 0.05 vs. IP group.

The administration of a triple dose of levamisole results in a significant increase in PDW at 72 h (*p* = 0.01) of inflammation compared to results obtained in the group with the IP group (Figure 3). All results are included in Table S1 (as Supplementary Data).

This experiment investigated the interdependence between hematological parameters and time to pleuritis onset (Table 1). The IP group demonstrated a moderately positive correlation (r = 0.533) between WBC count and time from the moment of carrageenan solution administration. No statistically significant temporal correlation between RBC, HGB, HCT, NEUT, LYM, and PLT and the initiated pleuritis was noted.

After administration of a single dose of levamisole, the LMS I dose group showed a strong positive correlation between the elapse of time and hemoglobin level (r = 0.776) as well as RBC count (r = 0.599). Single-dose administration of levamisole had an effect on HCT level (r = 0.471) as well as on WBC (r = 0.833) and PLT counts (r = 0.687). In the LMS I and III dose groups, respectively, a moderate (r = 0.648) and strong positive correlation (r = 0.734) between lymphocyte count and levamisole administration were observed.

Triple-dose administration of levamisole resulted in strong negative correlations concerning RBC count (r = −0.740), and hemoglobin (r = −0.771) and hematocrit (r = −0.777) levels, and in moderate negative correlations for neutrophil (r = −0.669) and platelets (r = −0.629) counts. LMS I group did not demonstrate a statistical correlation between levamisole administration and neutrophil count (*p* > 0.05), while in the LMS III group, no statistically significant correlation for white blood cell count (*p* > 0.05) was observed.

### 3.4. Histological Evaluation

**Figure 4:**
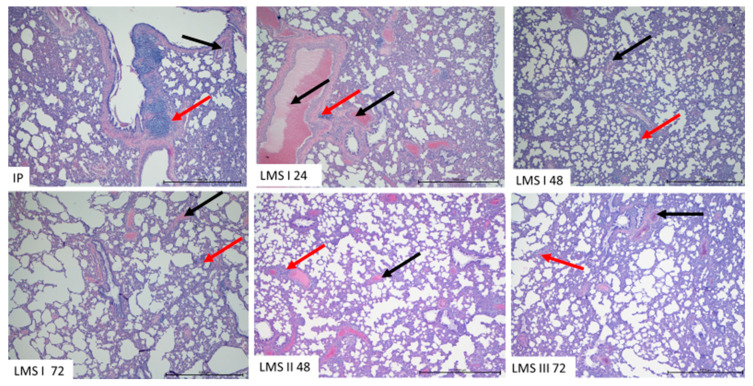
The representative pictures of lungs from animals of all experimental groups. Note the presence of the blood vessels filled with blood (black arrow) and leukocytic infiltration (red arrow) H&E. Mag. 200×. Scale bar—1000 µm.

**Figure 5:**
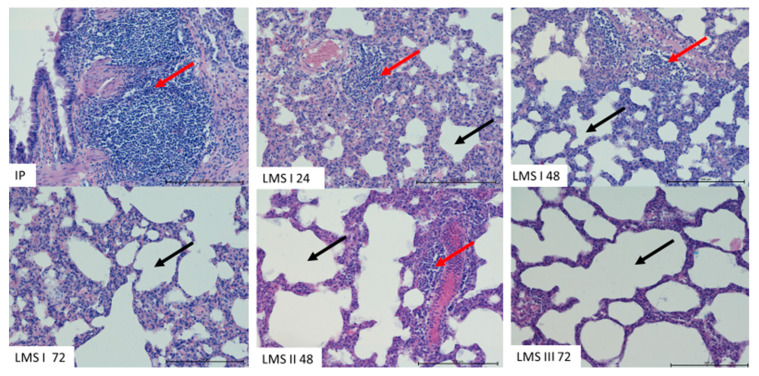
The representative pictures of lungs from animals of all experimental groups. Visible leukocytic infiltration in lungs (red arrow). The lumen of alveoli (black arrow) is not filled with fluids. H&E. Mag. 200×. Scale bar—200 µm.

In the IP group, after 72 h, strong infiltration of leukocytes was observed in the wall of bronchi and around blood vessels. The infiltration was composed mostly of macrophages and lymphocytes. Some neutrophils were noted in blood vessels. In the LMS I group, similar infiltrations were visible until 48 h, but after 72 h, the number of leukocytes in the lungs decreased. No oedemic changes in the lungs occurred; however, the vessels, both arteries and veins, were filled with blood. The lumen of bronchi and alveoli were filled with naturally occurring cells (Figure 4 and Figure 5). In LMS II 48 and LMS III 72, the decrease in inflammatory sites was more advanced, and lung tissue in multiple areas did not show advanced changes.

## 4. Discussion

The observed decrease in RBC count, hemoglobin and hematocrit levels should be explained by erythrocyte deficiency resulting from their entrapment at sites of inflammation, especially in lungs where high vascular capacity may be seen. Decreasing RBC count also depends on hemolysis. The above-mentioned result concerning the decrease in RBC count at 72 h after triple-dose administration of levamisole is correlated with reductions in hemoglobin and hematocrit levels. These data are not statistically significant; nevertheless, this tendency we have observed is unexpected and indicates that levamisole seems to not have an influence on these parameters.

Previous studies have shown that models of experimental inflammation may be associated with the development of disseminated intravascular coagulation (DIC), which in turn results in a decrease in the dynamics of RBC indices [27,28,29]. These results demonstrate the effect of a double and triple dose of levamisole on platelets. In addition, a single-dose administration of levamisole prevents a decrease in RBC count, hematocrit, and hemoglobin levels at 72 h.

A decrease in hemoglobin and hematocrit levels also results from increased levels of complement proteins (C3 and C4) that coat RBCs and thus reduce their resistance to hemolysis. In the same time interval, we observe an increase in haptoglobin and a decrease in transferrin levels, i.e., factors that play an important role in the preservation of hemoglobin iron from destroyed erythrocytes [27].

The administration of a single dose of levamisole stimulates thrombopoiesis and increases platelet count at 24 h and 72 h post-injection compared to the control pleuritis group at the same time points. However, triple-dose administration of levamisole inhibits platelet production and results in a significant decrease in platelet count, which in turn leads to plateletcrit reduction. The use of a triple dose of levamisole has a statistically significant effect of increasing PDW compared to the control inflammation group. As a result, levamisole impacts megakaryocytes in the course of thrombopoiesis.

At 24 h of inflammation, a decrease in leukocyte count compared to the physiological state is observed [30]. This situation should be explained by the migration of leukocytes to the site of inflammation when body compensation, normally occurring at 48 h of inflammation, is not present [25]. The use of a single dose of levamisole results in an increase in lymphocyte count at 24, 48 and 72 h of the experiment compared to the control inflammation model (at the same time points). Moreover, double and triple administration of levamisole has even more of an effect on the increase in the count of these white blood cell system components compared to the control inflammation model. This experimental model demonstrates that levamisole mainly stimulates a lymphocytic response. Both single- as well as multiple-dose administration results in lymphocyte stimulation. However, administration of a single dose of levamisole increases neutrophil count on day 3 of the experiment, while administration of three doses of levamisole has an immunosuppressive effect on the neutrophil count. However, in the lungs, histological examination revealed decreased intensity and number of sites of inflammation.

## 5. Conclusions

The use of a single dose of levamisole results in a change in hematological indices at 72 h post-administration, leading to an increase in RBC and platelet count and hemoglobin and hematocrit levels. On the other hand, the group receiving three doses of levamisole demonstrated a decrease in RBC and platelet count, as well as hemoglobin, hematocrit and plateletcrit levels.

In the course of the entire experiment, administration of a single dose of levamisole resulted in an increase in lymphocyte count at 24 h and 48 h post-pleurisy induction. In a group receiving three doses of levamisole, an intense increase in lymphocyte count was observed. Moreover, administration of a single dose of levamisole boosts the increase in the neutrophil count at 72 h, while administration of three doses of levamisole results in a decrease in the neutrophil count at 72 h.

## Figures and Tables

**Table 1:** Correlation coefficients r between hematological parameters and inflammation duration. Statistically significant dependencies are marked in bold.

*t* [h]	RBC	HGB	HCT	WBC	NEUT	LYM	PLT
IP	r = −0.256	r = −0.311	r = −0.259	**r = 0.533**	r = −0.137	r = 0.338	r = 0.256
	*p* = 0.305	*p* = 0.209	*p* = 0.300	***p* = 0.023**	*p* = 0.589	*p* = 0.170	*p* = 0.305
LMS I dose	**r = 0.599**	**r = 0.776**	**r = 0.471**	**r = 0.833**	r = −0.044	**r = 0.648**	**r = 0.687**
	***p* = 0.009**	***p* < 0.001**	***p* = 0.049**	***p* < 0.001**	*p* = 0.861	***p* = 0.004**	***p* = 0.002**
LMS III doses	**r = −0.740**	**r = −0.771**	**r = −0.777**	r = 0.301	**r = −0.669**	**r = 0.734**	**r = −0.629**
	***p* < 0.001**	***p* < 0.001**	***p* < 0.001**	*p* = 0.225	***p* = 0.002**	***p* = 0.001**	***p* = 0.005**

## Data Availability

Not applicable.

## Supplementary Materials

The following supporting information can be downloaded at: www.mdpi.com/article/10.3390/ani12162110/s1, Table S1: The mean values (X), standard deviation (SD) and *p*-values of selected hematological parameters between individual groups of rats with carrageenan-induced inflammation after single and triple levamisole application.
