# Supplementary material for: Effect of a Single and Triple Dose of Levamisole on Hematological Parameters in Controlled Inflammation Model
Source: Animals (Basel). 2022 Aug 17;12(16):2110. doi: 10.3390/ani12162110 (PMC9404755; doi:10.3390/ani12162110)
Supplement: Supplementary file 1 [file animals-12-02110-s001.zip › animals-1802468-supplementary.pdf]

Table S1. The mean values [X], standard deviation [SD] and *p* values of selected hematological parameters between individual groups of rats with carrageenan-induced inflammation after single and triple levamisole application.

Hematological parameter	time indicator	**IP**		**Levamisole one dose** (LMS I 24 h, LMS I 48 h, LMS I 72 h)			**Levamisole three doses** (LMS II 48 h, LMS III 72 h)		
		X	SD	X	SD	*p* (vs. IP)	X	SD	*p* (vs IP)
RBC ($x10^6/\mu L$)	24 h	6.26	1.93	5.68	0.19	0.52			
	48 h	6.23	0.78	5.55	0.78	0.16	5.37	0.23	0.04
	72 h	5.19	2.29	6.86	0.66	0.08	3.51	1.33	0.09
HGB (g/dL)	24h	11.50	3.62	10.40	0.56	0.52			
	48 h	11.16	1.15	10.26	0.54	0.11	9.56	0.62	0.02
	72 h	9.14	4.02	13.64	1.04	0.01	6.18	2.30	0.08
HTC (%)	24 h	33.54	10.09	34.72	1.62	0.80			
	48 h	34.08	2.21	34.53	2.11	0.73	31.84	1.39	0.07
	72 h	28.35	10.53	38.01	3.61	0.04	21.55	7.26	0.14
RDW (%)	24 h	15.10	2.54	16.58	0.83	0.23			
	48 h	16.23	1.05	16.45	0.80	0.45	16.02	1.37	0.76
	72 h	16.68	0.45	16.23	0.27	0.27	16.53	1.47	0.84
MCV (fL)	24 h	53.44	4.92	61.14	1.35	<0.001			
	48 h	55.17	4.16	60.11	0.63	0.01	59.34	0.52	0.05
	72 h	57.15	6.18	55.41	0.78	0.48	62.34	3.17	0.05
MCH (pg)	24 h	18.40	0.85	18.30	0.66	0.82			
	48 h	17.94	0.63	17.98	0.78	0.81	17.82	0.55	0.72
	72 h	17.65	0.61	19.91	0.55	<0.001	17.64	0.47	0.96
MCHC (g/dL)	24 h	34.69	0.23	29.90	0.86	<0.001			
	48 h	32.62	0.62	30.11	0.97	0.01	30.04	0.93	<0.001
	72 h	31.24	0.37	35.93	0.78	<0.001	28.33	1.11	0.02
PLT ($x10^3/\mu L$)	24 h	560.30	242.40	627.80	115.55	0.30			
	48 h	616.10	155.10	621.30	125.00	0.46	393.40	68.59	0.02
	72 h	672.80	158.80	905.57	76.75	0.01	359.13	199.75	<0.001
MPV (fL)	24 h	6.32	0.45	6.10	0.25	0.34			
	48 h	6.72	0.25	6.23	0.30	0.22	6.84	0.33	0.64
	72 h	6.70	0.16	6.54	0.06	0.14	6.58	0.35	0.60
PCT (%)	24 h	0.36	0.16	0.41	0.08	0.53			
	48 h	0.42	0.10	0.41	0.13	0.42	0.27	0.05	0.02
	72 h	0.45	0.10	0.42	0.21	0.11	0.24	0.13	<0.001
PDW (%)	24 h	57.18	3.45	60.06	1.77	0.11			
	48 h	55.67	3.81	58.23	3.21	0.23	56.74	2.26	0.58
	72 h	55.24	3.08	57.00	3.01	0.29	61.41	5.15	0.01
WBC ($x10^3/\mu L$)	24 h	3.74	2.52	6.56	0.31	0.03			
	48 h	5.76	1.36	6.98	0.25	0.05	8.07	1.12	0.01
	72 h	7.77	4.06	21.78	4.44	<0.001	7.93	3.10	0.93

HDW (g/dL)	24 h	2.26	0.15	2.06	0.08	0.16			
	48 h	4.60	2.81	2.01	0.98	<0.001	1.99	0.11	0.06
	72 h	2.14	0.31	2.11	0.33	0.27	1.99	0.11	0.21
NEUT ($x10^3/\mu L$)	24 h	1.99	1.59	3.26	1.09	0.14			
	48 h	2.89	0.28	3.16	0.55	0.13	2.72	0.93	0.78
	72 h	3.12	0.41	3.18	0.64	0.34	2.28	1.01	0.32
LYMPH ($x10^3/\mu L$)	24 h	1.49	1.04	2.83	0.76	0.02			
	48 h	1.80	0.89	2.96	0.82	<0.001	3.65	0.57	<0.001
	72 h	3.49	1.95	3.96	0.63	0.15	4.22	2.10	0.48
MONO ($x10^3/\mu L$)	24 h	0.41	0.03	0.79	0.06	0.11			
	48 h	0.56	0.27	0.77	0.18	0.04	0.59	0.07	0.42
	72 h	0.58	0.24	0.58	0.24	0.33	0.58	0.05	0.02
EOS ($x10^3/\mu L$)	24 h	0.09	0.01	0.09	0.01	0.26			
	48 h	0.18	0.03	0.08	0.01	0.01	0.20	0.02	0.25
	72 h	0.07	<0.001	0.21	0.02	<0.001	0.09	0.01	0.04
LUC ($x10^3/\mu L$)	24 h	0.19	0.02	0.37	0.04	<0.001			
	48 h	0.37	0.02	0.37	0.02	0.40	0.67	0.12	0.03
	72 h	0.50	0.03	0.65	0.05	0.02	0.62	0.04	0.02
BASO ($x10^3/\mu L$)	24 h	0.02	0.01	0.03	<0.001	0.01			
	48 h	0.02	0.12	0.03	<0.001	0.15	0.03	<0.001	0.17
	72 h	0.02	0.01	0.03	<0.001	0.10	0.02	<0.001	0.43
